# Case Report: Thymoma-associated myasthenia gravis, myositis, myocarditis, and anti-GAD65 autoimmune encephalitis: a unique case of paraneoplastic polyautoimmunity

**DOI:** 10.3389/fimmu.2026.1763320

**Published:** 2026-02-10

**Authors:** Enmanuel A. Leiva-Murillo, Eugenia Martínez-Hernández, Iban Aldecoa, Pedro Castro, Valeria Richart, José C. Milisenda, Ana Matas-García

**Affiliations:** 1 Muscle Research Unit, Department of Internal Medicine, Hospital Clínic, University of Barcelona, Barcelona, Spain; 2 Department of Neurology, Hospital Clínic, University of Barcelona, Neuroimmunology Program, Institut d’Investigacions Biomèdiques August Pi i Sunyer/CaixaResearch Institute, Barcelona, Spain; 3 Department of Pathology, Hospital Clínic, University of Barcelona, and Neurological Tissue Bank of the Biobank Fundació Clínic per a la Recerca Biomèdica (FCRB)/Institut d’Investigacions Biomèdiques August Pi i Sunyer (IDIBAPS), Hospital Clínic, Barcelona, Spain; 4 Medical Intensive Care Unit, Hospital Clínic-IDIBAPS, University of Barcelona, Barcelona, Spain; 5 Imaging Diagnostic Center, Department of Radiology, Hospital Clínic, University of Barcelona, Barcelona, Spain

**Keywords:** autoimmune encephalitis, inflammatory myopathy, myasthenia gravis, myocarditis, neuroimmunology, thymoma

## Abstract

Neurological and autoimmune muscle comorbidities are rare in thymoma-associated myasthenia gravis (TAMG). The incidence of myositis is likely underestimated due to its clinical similarity. Few cases of autoimmune encephalitis (AE) have been reported in TAMG, most of which are associated with neuronal surface antibodies. We present the case of a 41-year-old woman with weakness and bulbar dysfunction, and elevated muscle and cardiac enzyme levels, who developed seizures and a decreased level of consciousness. Among the complementary tests, electromyography revealed a postsynaptic neuromuscular junction disorder. Muscle biopsy revealed inflammatory myopathy (IM), and cardiac magnetic resonance imaging (MRI) showed myocardial edema. Electroencephalography showed epileptiform activity, while brain MRI revealed bilateral T2/FLAIR hyperintensities in the medial temporal lobe. Neuroimmunological studies detected anti-acetylcholine receptor and anti-glutamic acid decarboxylase 65 (anti-GAD65) antibodies. Chest computed tomography (CT) revealed a mediastinal mass, which was later confirmed as a thymoma. The patient received corticosteroids, intravenous immunoglobulin, plasma exchanges, and rituximab. The simultaneous coexistence of myasthenia, myositis, myocarditis, and AE in a patient with thymoma has not been previously described in the medical literature. The presence of anti-GAD65 in this context is exceptional. In this case, early recognition and aggressive treatment led to a remarkable recovery. Clinicians should maintain a high index of suspicion for the coexistence of autoimmune disorders in patients with TAMG. IM may indicate a more serious disease, and myocarditis can be life-threatening. Neurological signs, such as memory loss, confusion, and seizures, may indicate the development of AE.

## Introduction

Myasthenia gravis (MG) is strongly associated with thymic pathology; approximately 70% of cases are linked to thymic hyperplasia, and up to 15% are related to thymoma (1). Coexisting autoimmune diseases have been reported in 13–22% of MG patients (2, 3) and in 45% of those with thymoma, where they often manifest as paraneoplastic syndromes, present in up to 20% of cases (1). This association suggests shared pathophysiological mechanisms, including loss of central tolerance, breakdown of peripheral immune regulation, and hyperactivation of B and T lymphocytes (4).

Neurological and muscular autoimmune comorbidities are uncommon in thymoma-associated MG (TAMG). We present a unique case of simultaneous coexistence of myositis, myocarditis, and autoimmune encephalitis (AE) in TAMG, which has not been previously reported in the medical literature.

## Case presentation

A 41-year-old woman with relevant past medical history of sleeve gastrectomy for grade II obesity performed one year before admission, presented with progressive symptoms over one month of dysphagia for liquids, dysphonia, axial and proximal limb weakness, and binocular diplopia that worsened throughout the day. Physical examination revealed right eye exotropia without ptosis, weakness of the neck extensors and shoulder and pelvic girdle muscles (Medical Research Council Strength Score 4/5), and positive fatigability. No objective sensory alterations were observed. The initial laboratory tests revealed elevated muscle and cardiac enzyme levels (**Table 1**). Within 24 hours, she developed acute ventilatory failure with bilateral palpebral ptosis and severe tetraparesis, requiring admission to the intensive care unit for noninvasive mechanical ventilation. Pulmonary CT angiography ruled out pulmonary embolism and revealed a precardiac mediastinal mass suggestive of thymoma.

**Table 1:** Complementary test at diagnosis and during follow-up.

Determinations	At diagnosis	At discharge	At 6 months	Units	Reference value
Aldolase	139.9	6.9	6.4	IU/L	< 7.6
ALT	198	12	13	IU/L	< 40
AST	259	17	22	IU/L	< 40
CK	1300	48	65	IU/L	< 200
LDH	586	148	–	IU/L	< 234
TnI-US	2,196.0	11.4	28.3	ng/L	< 45
NT-proBNP	2,765	155	–	pg/mL	< 300
MSA	Negative	–	–	–	Negative
anti-AchR	69	–	17	Nmol	< 0.5
anti-MuSK	Negative	–	–	–	Negative
anti-VGCC	< 40	–	–	pmol/L	< 40
anti-LRP4	Negative	–	–	–	Negative
anti-GAD65 Serum^a^	13,988.6	–	4668.1	U/mL	< 1.0
anti-GAD65 CSF^a^	740	–	–	U/mL	< 1.0
Onconeuronal Antibodies^b^	Positive. Anti-Titin immunoreactivity.	–	–	–	Negative

ALT, alanine aminotransferase; anti-AchR, anti-acetylcholine receptor; anti-GAD65, autoantibodies against glutamic-acid-decarboxylase-65; anti-LRP4, low-density lipoprotein 4; anti-MuSK, muscle-specific tyrosine kinase antibody; anti-VGCC, anti-Voltage-Gated Calcium Channels; AST, aspartate aminotransferase; CK, creatine kinase; LDH, lactate dehydrogenase; MSA, myositis-specific autoantibody; NT-proBNP, N-terminal pro-brain natriuretic peptide; TnI-US, ultrasensitive troponin I.

^a.^anti-GAD65 antibodies (serum and CSF) were measured at the Centre de Diagnòstic Biomèdic (CDB), Hospital Clínic Barcelona, using a chemiluminescent immunoassay (CLIA) and reported in U/mL. The laboratory reference value is <1.0 U/mL (≥1.0 U/mL considered positive).

^b.^Onconeuronal antibodies correspond to the CDB Onco+GAD CSF panel, performed with immunohistochemistry screening and immunoblot confirmation, and reported qualitatively (positive/negative) according to laboratory reference criteria.

**Figure 1:**
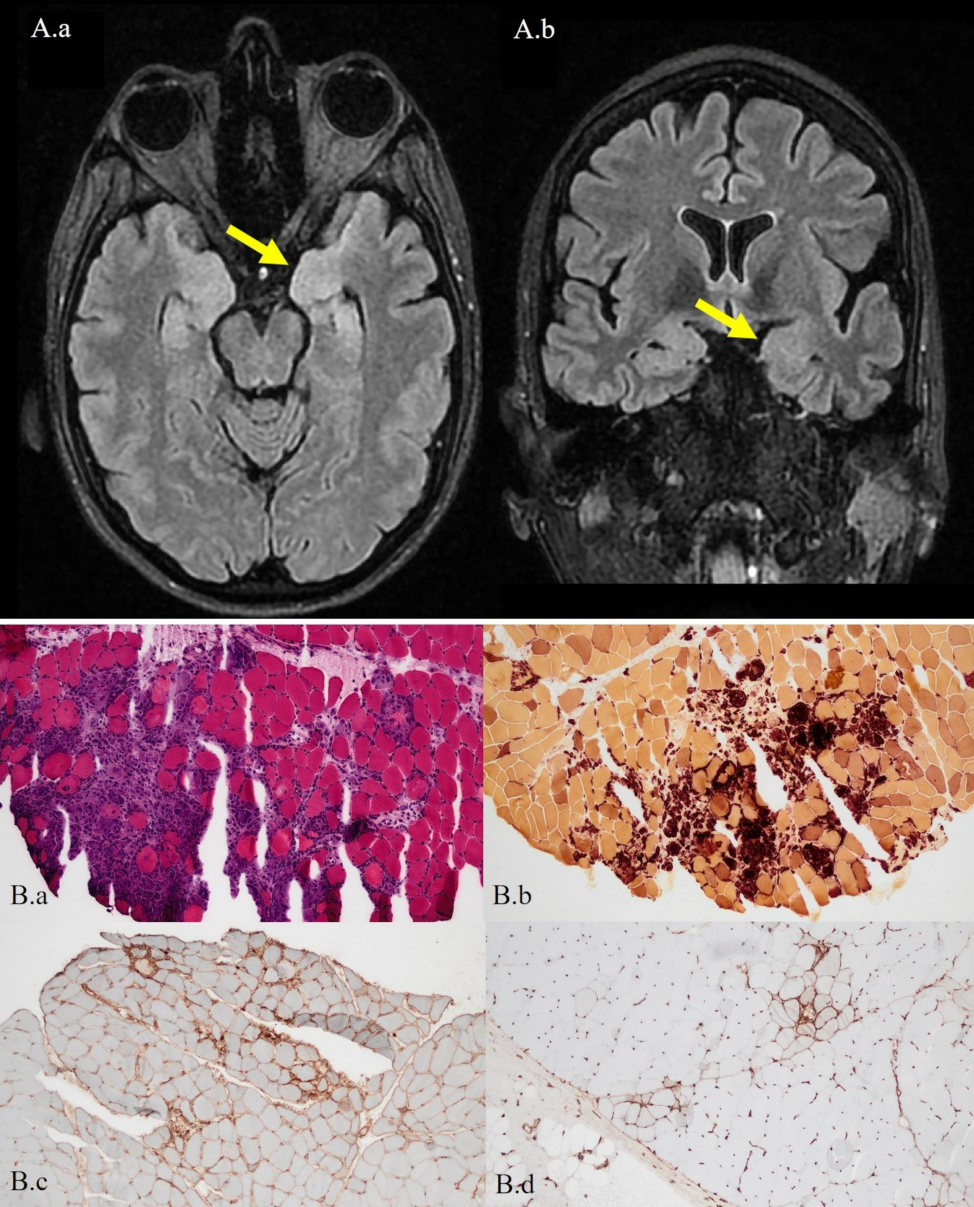
Brain MRI and muscle biopsy findings. **(A)** Brain MRI. Axial **(A.a)** and coronal **(A.b)** fluid-attenuated inversion recovery (FLAIR) images demonstrate subtle thickening and hyperintense signal in the hippocampal heads and amygdalae, more prominent on the left side (yellow arrow). **(B)** Muscle biopsy. **(B.a)** Hematoxylin and eosin staining showing dense inflammatory infiltrates with pseudogranulomatous involvement of the perimysium and endomysium. **(B.b)** Histochemical esterase staining highlighting a prominent monocytic infiltrate. **(B.c)** Immunohistochemistry for MHC class I showing diffuse sarcolemmal expression. **(B.d)** MHC class II immunohistochemistry revealing patchy positivity in the membrane, sarcoplasm, and sarcolemma. All muscle biopsy images were taken at 20× magnification.

**Figure 2:**
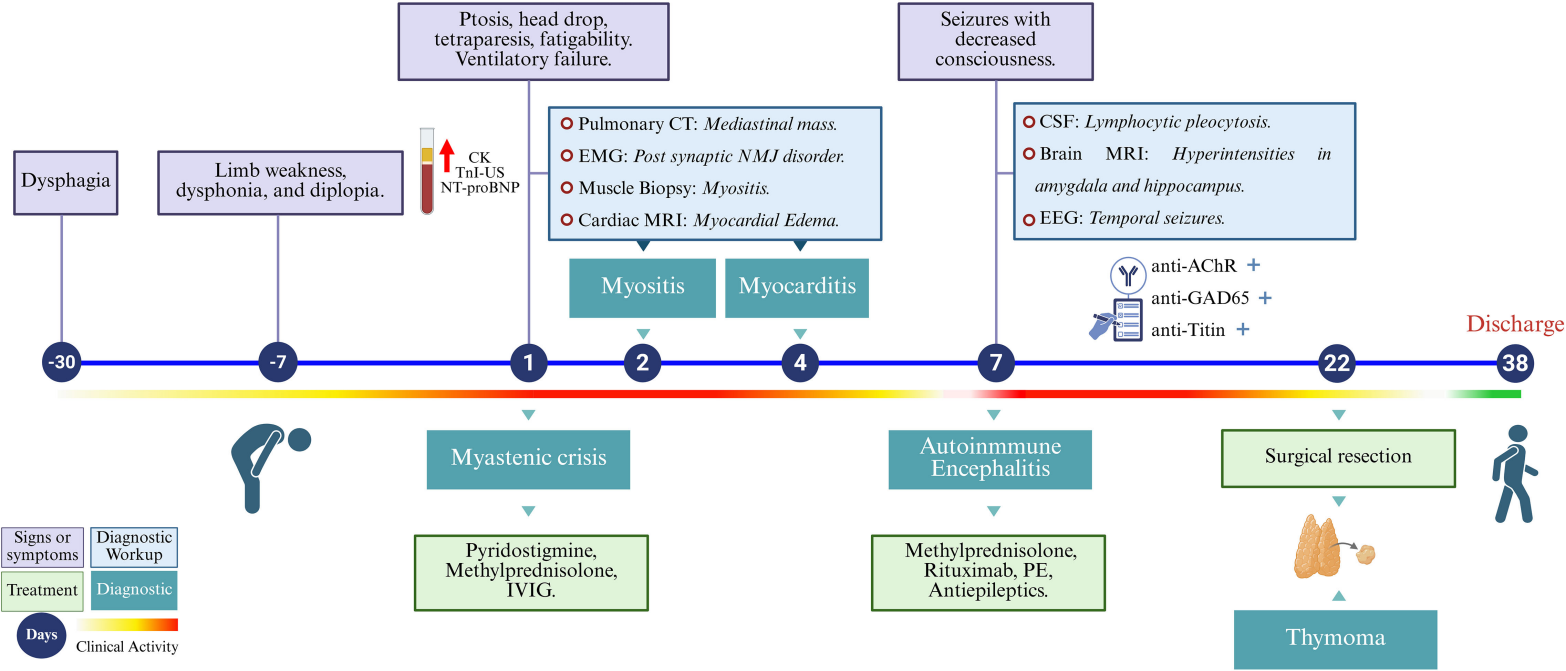
Clinical timeline of diagnosis and treatment. The timeline summarizes the patient’s clinical presentation, diagnostic workup, and treatment over a 38-day hospitalization. CK, creatine kinase; TnI-US, ultrasensitive troponin I; NT-proBNP, N-terminal pro-brain natriuretic peptide; CT, computed tomography; EMG, electromyography; MRI, magnetic resonance imaging; CSF, cerebrospinal fluid; EEG, electroencephalogram; anti-AchR, anti-acetylcholine receptor antibodies; anti-GAD65, anti–glutamic acid decarboxylase 65 antibodies; anti-Titin, anti-titin antibodies; IVIG, intravenous immunoglobulin; PE, plasma exchange.

A diagnosis of myasthenic crisis was established, and treatment with pyridostigmine, methylprednisolone pulses (1g/day for 3 days), and intravenous immunoglobulin (IVIG, 0.4 g/kg/d for 5 days) was initiated. Although ventilatory function improved, on day 7 of admission the patient developed a reduced level of consciousness (Glasgow Coma Scale 3/15) and generalized tonic-clonic seizures, requiring endotracheal intubation and invasive mechanical ventilation. Suspecting central nervous system involvement, a lumbar puncture was performed and cerebrospinal fluid (CSF) analysis revealed mild lymphocytic pleocytosis (10 WBC/mm3), without cytological or biochemical abnormalities; microbiological studies were negative. Brain MRI revealed bilateral T2/FLAIR hyperintensities in the amygdala and hippocampus (**Figure 1**), and electroencephalography (EEG) showed diffuse irregular slowing, alternating right interictal epileptiform activity, and left temporal focal seizures. Autoimmune limbic encephalitis (AE) was suspected, and immunological testing was expanded to detect antineuronal antibodies. Antiepileptic drugs were initiated, and immunotherapy was intensified with repeated methylprednisolone pulses, plasma exchange (PE), and rituximab, resulting in a favorable clinical response. The thymoma was surgically removed on day 22, and the patient was discharged from the hospital on day 38 (modified Rankin Scale score of 2). A graphical summary of the patient’s clinical evolution, ancillary tests, diagnoses, and therapeutic interventions is shown in the timeline (**Figure 2**).

## Diagnostic and therapeutic considerations

The initial differential diagnosis, based on the clinical symptoms and elevated muscle and cardiac enzyme levels, included severe myositis with bulbar and myocardial involvement versus myasthenia gravis (MG) with secondary myositis and myocarditis. The diagnosis of MG (5) was established based on compatible clinical features, high titers of anti-acetylcholine receptor (AChR) antibodies, and electromyographic evidence of decreased compound muscle action potential amplitudes, with a 14% decrement on 10 Hz repetitive stimulation and prolonged single-fiber jitter. The presence of anti-titin striational antibodies suggested an associated thymoma. Other relevant antibodies, including anti-ganglioside, antinuclear, and myositis-specific antibodies (MSAs), were negative. Muscle biopsy confirmed inflammatory myopathy (IM) with a polymyositis pattern (PM) and signs of acute denervation (**Figure 1**). Evidence of myocardial involvement included sinus tachycardia on electrocardiogram, dyspnea, elevated NT-proBNP, and increased troponin I. Transthoracic echocardiography revealed a preserved left ventricular ejection fraction (55%), segmental hypokinesia predominantly in the inferobasal wall, and diastolic dysfunction with a normal E/e′ ratio. No arrhythmias were detected on telemetry. Cardiac MRI demonstrated myocardial edema, with native T1 values of 1165 ms and T2 values ranging from 52–66 ms (normal 44 ± 3 ms), fulfilling current diagnostic criteria for definite myocarditis (6). In parallel, we evaluated alternative etiologies. An infectious myocarditis work-up was performed, and it was not supportive of active infection. Drug-, toxin-, or hypersensitivity-related myocarditis was considered, and no relevant exposures were identified. Structural and ischemic heart disease were also considered; however, imaging findings and the non-ischemic myocardial injury pattern on cardiac MRI made an acute coronary syndrome–type mechanism unlikely in this context.

Given the sudden neurological deterioration with reduced level of consciousness and seizures, the possibility of an acute central nervous system infection was quickly assessed through CSF analysis and microbiological studies, which were negative and only showed mild lymphocytic pleocytosis. Metabolic and toxic causes were evaluated through routine laboratory tests and clinical review, with no evidence of severe metabolic abnormalities or exposure to toxic substances. Acute structural or vascular aetiologies were evaluated through neuroimaging, which showed bilateral mesial temporal T2/FLAIR hyperintensities without other acute lesions. Seizure-related causes, including nonconvulsive status epilepticus, were evaluated by EEG, which confirmed temporal epileptiform activity and focal seizures, supporting an encephalitic process rather than an isolated postictal state. Taken together, these findings favored an autoimmune limbic process; accordingly, the patient was diagnosed with autoimmune limbic encephalitis (AE) (7), and high titers of anti-GAD65 antibodies were detected in both serum and CSF (**Table 1**).

The patient underwent robot-assisted thymectomy, and histopathological examination confirmed a B2 thymoma (Masaoka stage IIA, T1N0M0). Confirmed diagnoses included generalized MG, IM, myocarditis, AE, and thymoma. The clinical course was favorable, with resolution of respiratory, bulbar, and ocular symptoms, normalization of muscle and cardiac enzyme levels, and complete resolution of myocardial edema on follow-up cardiac MRI. Upon discharge, a tapering regimen of corticosteroids was prescribed, along with monthly plasma exchanges, and rituximab every six months, combined with pyridostigmine and antiepileptic drugs. Due to the thymoma-associated lymphovascular invasion, delayed adjuvant radiotherapy was initiated a few months later. Follow-up EEGs showed a progressive reduction in epileptiform activity; however, the patient remained with mild cognitive impairment. Serum anti-GAD65 antibodies remained positive, but their titers decreased significantly from initial levels (**Table 1**). At the 12-month evaluation, the patient had no muscle weakness or bulbar dysfunction and a favorable functional outcome (modified Rankin Scale score of 1). Follow-up brain MRI showed persistent bilateral T2/FLAIR hyperintensities in the amygdala and hippocampus, with no new lesions. A single focal epileptic discharge in the left frontotemporal region was recorded on the EEG, with no clinical manifestations.

## Discussion

We present a unique case of simultaneous coexistence of myasthenia, myositis, myocarditis, and autoimmune encephalitis associated with thymoma, a combination not previously reported. To place this presentation in context, thymoma-driven breakdown of central tolerance provides a unifying framework for multi-organ autoimmunity.

The thymus is a primary lymphoid organ where key immunotolerance processes occur, including the negative selection of autoreactive T cells and the generation of regulatory T cells. These tolerogenic mechanisms, which are partly dependent on the AIRE (autoimmune regulator) transcription factor, help prevent the development of autoimmunity. The characterization of inflammatory and neoplastic thymic disorders in myasthenia gravis has enabled the delineation of central pathways linking thymic dysfunction to autoimmune phenomena (8).

Thymomas are tumors of thymic epithelial cells, which can maintain active thymopoiesis. However, the disorganized tumor architecture and reduced expression of molecules involved in central tolerance, such as AIRE and the major histocompatibility complex class II, together with decreased production of regulatory T cells, promote the development of autoreactive T lymphocytes that escape apoptosis and are capable of stimulating B cells in the periphery to generate autoantibodies against AChR and other muscle antigens. This is due to the absence of thymic myoid cells or the aberrant expression of muscle epitopes in the neoplastic thymic epithelium (8, 9).

This framework is particularly relevant in type B2 thymoma, characterized by an abundant population of immature lymphocytes and cortical-type neoplastic epithelial cells, a subtype most frequently linked to autoimmune and paraneoplastic phenomena (9).

IM has been reported in only 1-2.9% (10, 11) of MG patients, with approximately 50 cases documented (10). In TAMG, IM has been linked to antibodies against striated muscle proteins, such as titin, ryanodine receptor (RyR), and voltage-gated potassium channels (Kv1.4) (12, 13). Although the pathogenicity of these antibodies remains uncertain, their presence correlates with more frequent myasthenic crises, earlier progression to generalized MG, and greater bulbar involvement (14). An alternative theory postulates that thymoma-driven immune dysregulation, similar to that observed in immune checkpoint inhibitor–associated IM, may contribute to pathogenesis. This hypothesis is supported by findings of PD-1-positive endomysial cells and overexpression of PD-L1 and CTLA4 in muscle tissue (11). In addition, it is likely that circulating MG-related antibodies facilitate recruitment of autoreactive T cells to the muscle surface via interaction with postsynaptic neuromuscular junction receptors (15).

As in our case, IM usually presents simultaneously with MG and is associated with thymoma in more than half of the reported cases. The most common histological pattern is PM, characterized by CD8+ T-cell infiltration (11), although dermatomyositis and granulomatous myopathy have also been described (10). IM in TAMG is often MSA-negative, as observed in our patient. Myocardial involvement may share a similar pathophysiological basis, and mounting evidence supports a role for anti-striated muscle antibodies in cardiac tissue reactivity (12, 13). A recent systematic review reported 35 cases of MG-associated myocarditis, nearly half of which occurred in the context of immunotherapy. Myocarditis was present at MG diagnosis in 37% of cases, was more frequent in males (57%), and carried a mortality rate exceeding 50% among symptomatic patients, with dyspnea as the most common presenting symptom (16).

Since its first description in 1988, several cases of thymoma-associated AE have been reported. In a recent series of 43 patients with thymoma and AE, 90% had at least one neuronal surface antibody, and 30% had intracellular antibodies, with anti-GAD65 positivity in 3 cases. The clinical presentation was heterogeneous, and neither the presence of MG (30%) nor advanced thymoma (50%) correlated with the encephalitis subtype or antibody profile. Only six patients met the criteria for limbic encephalitis, and none were anti-GAD65-positive (17). Regarding TAMG-associated AE, at least 18 cases have been reported. Most occurred in women (60%) around age 44, with MG and AE diagnosed simultaneously in at least half. Ocular muscles were most commonly affected (83%), followed by bulbar (50%) and respiratory (27%) involvement; cervical (11%) and facial (5.5%) involvement were less frequent. Neurological features typically included memory loss, confusion, and seizures. Brain MRI abnormalities, predominantly affecting the hippocampus and temporal lobes, were observed in over half of the patients. Antibody profiles showed anti-AChR positivity in 94%, anti-titin in 22%, and encephalitis–related antibodies in 70%, most commonly AMPAR and CASPR2 or LGI1 (those previously recognized as anti-VGKC) (18). Our case shared many of these clinical features but uniquely tested positive for anti-GAD65 antibodies. Anti-GAD65 AE is a subgroup of limbic encephalitis that typically affects young women with prominent seizures, usually without underlying malignancy (19). Nonetheless, as in our case, paraneoplastic associations have been described, particularly when anti-GAD65 coexists with neuronal surface antibodies or when clinical presentation is atypical. Lung and thymic neoplasms are the most frequently associated tumors (20). Early identification of anti-GAD65 AE has prognostic implications, as delayed initiation of immunotherapy is associated with worse outcomes. Severe clinical forms such as refractory status epilepticus or cerebellar ataxia, limbic involvement, and persistent neuroimaging abnormalities are factors associated with poor prognosis (21). No clear correlation has been established between antibody levels and disease severity or seizure persistence; however, reductions in anti-GAD65 levels have been observed in patients with seizure improvement after treatment initiation, although complete antibody elimination is rare (22).

Although in other paraneoplastic syndromes associated with anti-GAD65, the presence of thymoma seems to be associated with a better prognosis (18, 20), in cases of encephalitis, this relationship is not consistent, with cases of clinical progression after thymectomy in this subgroup, which contrasts with the good response to immunotherapy and surgery observed in other autoimmune encephalitis associated with thymoma and myasthenia gravis (21).

Evidence indicates that anti-GAD65 tumor-associated encephalitis responds poorly to conventional immunotherapy, so in severe, refractory, or recurrent cases, intensification and long-term maintenance of immunosuppression may be considered to control residual autoimmune activity and mitigate the risk of relapse, with the overall goal of minimizing progressive neurological damage and optimizing functional outcomes, even when anti-GAD65 titers remain detectable (22). Given the limited empirical basis and the absence of randomized clinical trials, the therapeutic strategy must be individualized. In this context, and given our patient’s severe, multi-organ autoimmune phenotype, we chose monthly plasma exchange as a complementary strategy and rituximab as maintenance therapy to reduce the risk of relapse and preserve functional outcomes despite the persistence of anti-GAD65 positivity.

In the case we present, timely recognition of a clinical picture suggestive of autoimmune encephalitis, together with early and intensive immunotherapy and a favorable initial response, were probably decisive factors in the good long-term results obtained.

## Conclusions

The simultaneous occurrence of MG, IM, myocarditis, and AE in thymoma is exceptionally rare, and, to our knowledge, may be one of the first cases of TAMG with anti-GAD65 autoimmune limbic encephalitis. The true incidence of IM in patients with MG is likely underestimated due to overlapping clinical features, infrequent enzyme testing, and limited use of muscle biopsy. In patients with MG, the presence of IM may indicate a more severe disease course, with increased risk of bulbar involvement, myasthenic crises, and myocarditis, the latter being potentially life-threatening. Clinicians should maintain a high index of suspicion for the coexistence of autoimmune disorders in patients with MG who present with atypical clinical features, such as confusion, memory loss, or seizures, particularly in the setting of thymoma. These symptoms may signal the development of AE. Although there is no standardized treatment approach, our case highlights the importance of a multidisciplinary strategy and early, intensive immunotherapy, particularly in patients with bulbar involvement, myocarditis, and AE.

## Data Availability

The original contributions presented in the study are included in the article/supplementary material, Further inquiries can be directed to the corresponding author/s.

## References

[1] Holbro A Jauch A Lardinois D Tzankov A Dirnhofer S Hess C . High prevalence of infections and autoimmunity in patients with thymoma. Hum Immunol. (2012) 73:287–90. doi: 10.1016/j.humimm.2011.12.022, PMID: 22261388

[2] Mao ZF Yang LX Mo XA Qin C Lai YR He NY . Frequency of autoimmune diseases in myasthenia gravis: a systematic review. Int J Neurosci. (2011) 121:121–9. doi: 10.3109/00207454.2010.539307, PMID: 21142828

[3] Fang F Sveinsson O Thormar G Granqvist M Asking J Lundberg IE . The autoimmune spectrum of myasthenia gravis: a Swedish population-based study. J Intern Med. (2015) 277:594–604. doi: 10.1111/joim.12310, PMID: 25251578

[4] Nacu A Andersen JB Lisnic V Owe JF Gilhus NE . Complicating autoimmune diseases in myasthenia gravis: a review. Autoimmunity. (2015) 48:362–8. doi: 10.3109/08916934.2015.10306147, PMID: 25915571 PMC4616023

[5] Gilhus NE Tzartos S Evoli A Palace J Burns TM Verschuuren JJGM . Myasthenia gravis. Nat Rev Dis Primers. (2019) 5:1–19. doi: 10.1038/s41572-019-0079-y, PMID: 31048702

[6] Martens P Cooper LT Tang WHW . Diagnostic approach for suspected acute myocarditis: considerations for standardization and broadening clinical spectrum. J Am Heart Assoc. (2023) 12:e031454. doi: 10.1161/JAHA.123.031454, PMID: 37589159 PMC10547314

[7] Graus F Titulaer MJ Balu R Benseler S Bien CG Cellucci T . A clinical approach to diagnosis of autoimmune encephalitis. Lancet Neurol. (2016) 15:391–404. doi: 10.1016/S1474-4422(15)00401-9, PMID: 26906964 PMC5066574

[8] Marx A Yamada Y Simon-Keller K Schalke B Willcox N Ströbel P . Thymus and autoimmunity. Semin Immunopathol. (2021) 43:45–64. doi: 10.1007/s00281-021-00842-3, PMID: 33537838 PMC7925479

[9] Marx A Willcox N Leite MI Chuang W-Y Schalke B Nix W . Thymoma and paraneoplastic myasthenia gravis. Autoimmunity. (2010) 43:413–27. doi: 10.3109/08916930903555935, PMID: 20380583

[10] Garibaldi M Fionda L Vanoli F Leonardi L Loreti S Bucci E . Muscle involvement in myasthenia gravis: expanding the clinical spectrum of myasthenia-myositis association from a large cohort of patients. Autoimmun Rev. (2020) 19:102498. doi: 10.1016/j.autrev.2020.102498, PMID: 32062029

[11] Uchio N Taira K Ikenaga C Kadoya M Unuma A Yoshida K . Inflammatory myopathy with myasthenia gravis: thymoma association and polymyositis pathology. Neurol Neuroimmunol Neuroinflamm. (2019) 6:e535. doi: 10.1212/NXI.0000000000000535, PMID: 30697585 PMC6340335

[12] Suzuki S . Autoimmune targets of heart and skeletal muscles in myasthenia gravis. Arch Neurol. (2009) 66:1334. doi: 10.1001/archneurol.2009.229, PMID: 19752287

[13] Suzuki S Baba A Kaida K Utsugisawa K Kita Y Tsugawa J . Cardiac involvements in myasthenia gravis associated with anti-Kv1.4 antibodies. Eur J Neurol. (2014) 21:223–30. doi: 10.1111/ene.12234, PMID: 23829303

[14] Chen Y Tao X Wang Y Xu S Yang Y Han J . Clinical characteristics and prognosis of anti-AChR positive myasthenia gravis combined with anti-LRP4 or anti-titin antibody. Front Neurol. (2022) 13:873599. doi: 10.3389/fneur.2022.873599, PMID: 35614931 PMC9124862

[15] Pinal-Fernandez I Quintana A Milisenda JC Casal-Dominguez M Muñoz-Bracenas S Derfoufi A . Transcriptomic profiling reveals distinct subsets of immune checkpoint inhibitor induced myositis. Ann Rheum Dis. (2023) 82:829–36. doi: 10.1136/ard-2022-223792, PMID: 36801811 PMC10545139

[16] Cheng W Sun T Liu C Zhou Z Duan J Zhao Y . A systematic review of myasthenia gravis complicated with myocarditis. Brain Behav. (2021) 11:e2242. doi: 10.1002/brb3.2242, PMID: 34105901 PMC8413805

[17] Guasp M Landa J Martinez-Hernandez E Sabater L Iizuka T Simabukuro M . Thymoma and autoimmune encephalitis: clinical manifestations and antibodies. Neurol Neuroimmunol Neuroinflamm. (2021) 8:e1053. doi: 10.1212/NXI.0000000000001053, PMID: 34301822 PMC8312280

[18] Su M Luo Q Wu Z Feng H Zhou H . Thymoma-associated autoimmune encephalitis with myasthenia gravis: case series and literature review. CNS Neurosci Ther. (2024) 30:e14568. doi: 10.1111/cns.14568, PMID: 38421083 PMC10850820

[19] Malter MP Helmstaedter C Urbach H Vincent A Bien CG . Antibodies to glutamic acid decarboxylase define a form of limbic encephalitis. Ann Neurol. (2010) 67:470–8. doi: 10.1002/ana.21917, PMID: 20437582

[20] Ariño H Höftberger R Gresa-Arribas N Martínez-Hernández E Armangue T Krue MC . Paraneoplastic neurological syndromes and glutamic acid decarboxylase antibodies. JAMA Neurol. (2015) 72:874. doi: 10.1001/jamaneurol.2015.0749, PMID: 26099072 PMC4838033

[21] Kuang Z Baizabal-Carvallo JF Alonso-Juarez M Mofatteh M Rissardo JP Pan M . The limbic and extra-limbic encephalitis associated with glutamic acid decarboxylase (GAD)-65 antibodies: an observational study. Neurol Sci. (2025) 46:2765–77. doi: 10.1007/s10072-024-07933-7, PMID: 39704979

[22] Daif A Lukas RV Issa NP Javed A VanHaerents S Reder AT . Antiglutamic acid decarboxylase 65 (GAD65) antibody-associated epilepsy. Epilepsy Behav. (2018) 80:331–6. doi: 10.1016/j.yebeh.2018.01.021, PMID: 29433947

